# High-Performance Photodiode-Type Photodetectors Based on Polycrystalline Formamidinium Lead Iodide Perovskite Thin Films

**DOI:** 10.1038/s41598-018-29147-6

**Published:** 2018-07-24

**Authors:** Meng Zhang, Fan Zhang, Yue Wang, Lijie Zhu, Yufeng Hu, Zhidong Lou, Yanbing Hou, Feng Teng

**Affiliations:** Key Laboratory of Luminescence and Optical Information, Ministry of Education, Institute of Optoelectronic Technology, Beijing Jiaotong University, Beijing, 100044 China

## Abstract

Photodetectors based on three dimensional organic–inorganic lead halide perovskites have recently received significant attention. As a new type of light-harvesting materials, formamidinium lead iodide (FAPbI_3_) is known to possess excellent optoelectronic properties even exceeding those of methylammonium lead iodide (MAPbI_3_). To date, only a few photoconductor-type photodetectors based on FAPbI_3_ single crystals and polycrystalline thin films in a lateral structure have been reported. Here, we demonstrate low-voltage, high-overall-performance photodiode-type photodetectors in a sandwiched geometry based on polycrystalline α-FAPbI_3_ thin films synthesized by a one-step solution processing method and post-annealing treatment. The photodetectors exhibit a broadband response from the near-ultraviolet to the near-infrared (330–800 nm), achieving a high on/off current ratio of 8.6 × 10^4^ and fast response times of 7.2/19.5 μs. The devices yield a photoresponsivity of 0.95 AW^−1^ and a high specific detectivity of 2.8 × 10^12^ Jones with an external quantum efficiency (EQE) approaching 182% at −1.0 V under 650 nm illumination. The photodiode-type photodetectors based on polycrystalline α-FAPbI_3_ thin films with superior performance consequently show great promise for future optoelectronic device applications.

## Introduction

Photodetectors have attracted significant attention that find extensive applications in the fields such as optical communications, video imaging, environmental monitoring, chemical/biological sensing, and space exploration^1^. As an optoelectronic device converting a light signal into a detectable electrical signal, it is highly desirable that a broadband photodetector operating at low voltages should possess high photoresponsivity and detectivity with a fast response and high ratio of photocurrent to dark current^2–6^. Since the first report on the solar cells based on methylammonium lead iodide (MAPbI_3_) perovskite thin films^7^, tremendous efforts have been devoted to improving the performance of perovskite solar cells due to the extraordinary physical properties of this new generation of light-harvesting materials, such as appropriate direct bandgap, high absorption coefficient, wide absorption spectrum, large carrier mobility, and long carrier diffusion length^8^. Meanwhile, three-dimensional organic–inorganic hybrid perovskites have been extensively studied and applied to fabricating other optoelectronic devices^9^, especially photodetectors^10–17^, which adopt a general formula ABX_3_, where A is an methylammonium (MA or CH_3_NH_3_^+^) or formamidinium (FA or NH_2_CH = NH_2_^+^) cation, B is a metal ion (Pb^2+^ or Sn^2+^), and X is a halide anion (Cl^−^, Br^−^or I^−^)^18^.

Compared to MAPbI_3_, the larger FA cation in a FAPbI_3_ perovskite occupies the A site in the ABX_3_ perovskite structure, forming a more symmetric crystal structure and reducing the electronic band gap^19,20^. FAPbI_3_ perovskites also show wider absorption spectrum and better thermal stability^21–23^. They are consequently regarded as a more proper candidate material for light harvesting not only in solar cells, but also in photodetectors. Currently, most studies of FAPbI_3_ perovskites have been focused on FAPbI_3_-based solar cells^20^ and some on nanowire lasers of FAPbX_3_^24^ and light emitting diodes based on FAPbBr_3_^25^. Nevertheless, only a few photodetectors based on FAPbI_3_ single crystals and polycrystalline thin films^26–28^ have been reported. Additionally, a critical issue in synthesizing FAPbI_3_ perovskites is the phase purity since FAPbI_3_ materials have two phases. One is a desirable semiconducting perovskite phase (α-phase), and the other is an insulating non-perovskite phase (δ-phase) that is preferentially formed at room temperature^29–31^. It is therefore particularly important to promote the formation of α-FAPbI_3_ for achieving high performance in FAPbI_3_-based optoelectronic devices.

The performance parameters and operating mechanisms of perovskite photodetectors are determined by the device configuration as well as the optoelectronic properties of the photoactive perovskite material. Two major types of two-terminal photodetectors based on organic–inorganic hybrid perovskites: photoconductors in a lateral geometry^10,14,32^ and photodiodes in a sandwiched structure^11–13^, have been reported. Many MAPbI_3_ photoconductors have adopted a lateral structure and operated at relatively high voltages^33^, even as high as 10 V^34^, although some of them have demonstrated an external quantum efficiency (EQE) larger than 100%^35^. Meanwhile, they suffer from slow response that the rise time ranging from 0.02 ms to approximately 100 ms which limits their further applications in high-speed devices. The lateral photodetectors based on FAPbI_3_ single crystals^28^ and FAPbI_3_ polycryatalline thin films^26^ have also been demonstrated. The FAPbI_3_ crystal photodetector working at 0.1 V has response rise/fall times of 12.4/17.8 ms, even slower than those (5.4/10.9 ms) of the FAPbI_3_ film device at 10 V, while no EQE values have been reported in the two literatures. Photodiode-type photodetectors apparently operate at relatively low voltages and exhibit fast response owing to rather short lengths of the photoactive layers, but their EQE values are normally less than unity^13,36,37^, except that in some cases current amplification or photomultiplication has been realized by the interface-controlled charge injection and photoconductive gain^38–40^. The response times of the sandwiched photodetectors based on MAPbI_3_ are in the range from a few microseconds to tens of microseconds^11,37,41^. In contrast to MAPbI_3_, few reports on photodiode-type photodetectors based on FAPbI_3_ perovskites have been published so far.

In this paper, we demonstrate photodiode-type photodetectors based on polycrystalline α-FAPbI_3_ thin films. Pure polycrystalline α-FAPbI_3_ thin films were prepared using a one-step solution processing method and post-annealing treatment. Nanocrystal titanium dioxide (TiO_2_) and 2,2′,7,7′- Tetrakis[N,N-di(4-Methoxyphenyl)aMino] −9,9′-spirobifluorene (Spiro-OMeTAD) were utilized respectively as electron extraction/hole blocking and hole extraction/electron blocking layers to transport charge carriers and suppress dark current. The photodetectors exhibit superior performance and their operating mechanism are discussed.

## Results and Discussion

**Figure 1:**
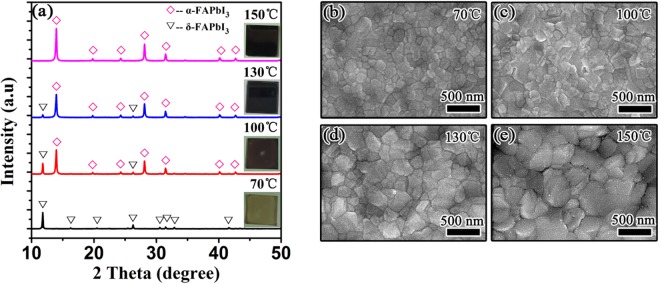
(**a**) XRD patterns and (**b**–**e**) top-view SEM images of the FAPbI_3_ films annealed in a nitrogen-filled glove box at different temperatures. The photographs of the FAPbI_3_ films are illustrated in the inset of Fig. 1(a).

Charge carrier transport in a perovskite photodetector is closely related to the crystallographic structure and morphology of the photoactive material, which impacts the performance parameters of the device^22,42^. We firstly investigated the effects of the post-annealing treatment on the crystallographic structures and morphologies of the FAPbI_3_ thin films. Figure 1(a) shows the XRD patterns of the FAPbI_3_ films which were spin coated on glass substrates and annealed in a nitrogen-filled glove box at temperatures of 70, 100, 130, and 150 °C, respectively. For the FAPbI_3_ film annealed at 70 °C, all the peaks can be indexed to the reflections of a polycrystalline hexagonal δ-phase FAPbI_3_. The strongest diffraction peak at 11.8° and the second strongest at 26.3° correspond to the (010) and (021) planes of the δ-FAPbI_3_ thin film, respectively^28,29^. As the annealing temperature increases to 100 °C, the intensities of the two dominant peaks at 11.8° and 26.3° for the δ-phase become smaller and the other weaker peaks almost vanish. However, the (111), (222), and (123) reflection peaks of a trigonal FAPbI_3_ phase (α-phase) appear at 13.9°, 28.1°, and 31.5°. The typical peaks of the α*-*phase perovskite further enhances as the temperature increases to 130 °C, while those of the δ*-*phase perovskite almost disappear. The pure α*-*phase FAPbI_3_ thin film has been obtained at the annealing temperature of 150 °C, which can be seen from the XRD pattern. The peaks at 13.9°, 19.8°, 24.3°, 28.1°, 31.5°, 40.2° and 42.8° can be assigned to the reflections of to the (111), (012), (021), (222), (123), (024) and (333) planes of the α*-*FAPbI_3_ film, respectively, agreeing well with those in previous reports^29,42^. Moreover, it is apparent that the α*-*phase polycrystalline FAPbI_3_ film exhibits (111) preferred orientation and a high degree of crystallinity, which facilitates charge carrier transport. The insets of Fig. 1(a) display the photographs of the FAPbI_3_ films annealed at different temperatures. We can find that the color of the film changes from yellow to black gradually with increasing temperature, which is also indicative of a complete transformation from the yellow δ-phase to the black α-phase. Figure 1(b–e) illustrate the top-view SEM images of the FAPbI_3_ thin films with annealing treatments at various temperatures. Obviously, the annealing temperature affects not only the phase purity but the morphologies of the FAPbI_3_ films. It is found that the films are compact, whereas the average grain size of the film increases with the annealing temperature. The pure α-FAPbI_3_ film annealed at 150 °C has the largest grain sizes between 200 and 300 nm, similar to those of the FAPbI_3_ films in the solar cells reported in the literature^22^, which are beneficial to the suppression of charge carrier recombination due to a decrease in grain boundaries.

**Figure 2:**
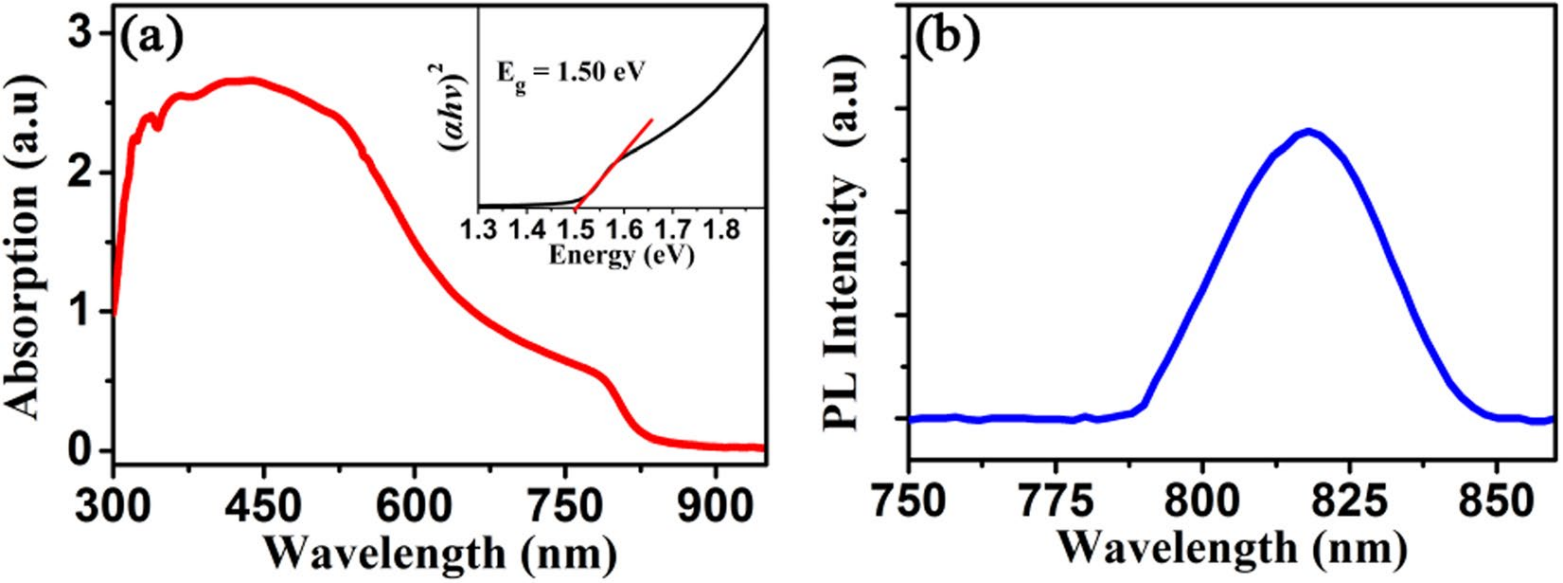
(**a**) UV absorption and (**b**) steady-state PL spectra of the α-FAPbI_3_ film annealed at 150 °C. The excitation wavelength for PL is 500 nm. The inset in Fig. 2(a) is the Tauc plot of the UV absorption spectrum.

The perovskite layer is a core material in a perovskite photodetector, the optical properties of which are of critical importance in determining the device performance. We studied the optical properties of the pure α-FAPbI_3_ thin film synthesized by the post annealing treatment at 150 °C. Figure 2(a) depicts the ultraviolet-visible (UV-Vis) absorption spectrum of the α-FAPbI_3_ film. The perovskite film presents a broad absorption spectrum covering the near-ultraviolet (n-UV), visible, and part of the near-infrared (NIR) regions with the absorption cut-off edge at about 825 nm, which is similar to that of α-FAPbI_3_ films^22,37^. For a direct bandgap semiconductor, the optical bandgap *E*_g_ can be derived by the Kubellka–Munk equation on the basis of the absorption spectrum^43^, which can be expressed as:

1 $${(\alpha h\upsilon )}^{2}=A(h\upsilon -{E}_{g})$$

where *α* is the absorption coefficient, *hv* is the incident photon energy, and *A* is a constant. The Tauc plot of the absorption spectrum for the α-FAPbI_3_ film is presented in the inset of Fig. 2(a). The optical bandgap of the film is estimated to be 1.50 eV by extrapolating the linear part of (*αhν*)2 versus photon-energy plot. The steady-state photoluminescence (PL) spectrum of the perovskite film is illustrated in Fig. 2(b). The PL peak is observed to be centered at 818 nm in accordance with the absorption onset at 825 nm in Fig. 2(a), suggesting a very low trap state density in the film that favors carrier transport^27^.

**Figure 3:**
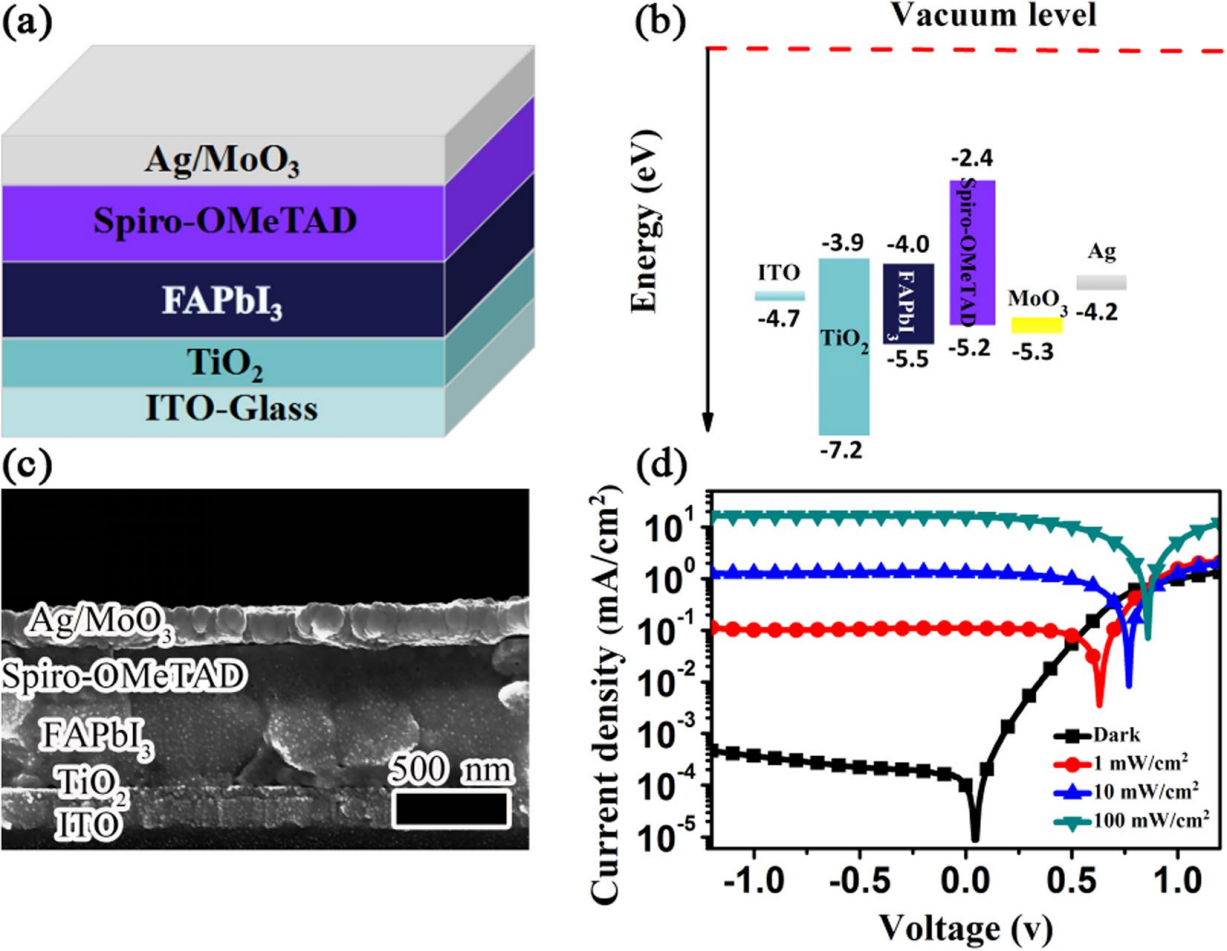
(**a**) Schematic diagram of the photodetector structure based on the pure α-FAPbI_3_ film. (**b**) Energy level diagram and (**c**) cross-sectional SEM image of the perovskite photodetector. (**d**) Current density-voltage curves of the photodetector in the dark and under white light illumination of different irradiances.

We applied the pure α*-*FAPbI_3_ polycrystalline film to fabricating a photodetector with a configuration of indium tin oxide (ITO)/TiO_2_/α*-*FAPbI_3_/Spiro-OMeTAD/molybdenum trioxide(MoO_3_)/silver(Ag), as shown in Fig. 3(a). Herein, the ITO and MoO_3_-modified Ag films served as the cathode and the anode, respectively. The thin film of anatase TiO_2_ nanocrystals was utilized as the electron extraction/hole blocking layer, which is not only used in polymer solar cells^44^ but also in organic photodetectors^45^ due to its high electron mobility, large bandgap, high transparency, and good chemical and thermal stability. The XRD peaks of the TiO_2_ layer, as shown in Figure S1 (Supplementary Information), are completely in conformity with those of JCPDS card file no. 21–1272 TiO_2_^44,46^, suggesting that TiO_2_ has the anatase phase. Figure S2 displays the atomic force microscopic (AFM) image of the TiO_2_ film. The film shows a smooth surface with a root-mean-square (RMS) roughness of 1.28 nm, which is beneficial to the crystallization of the FAPbI_3_ film. Spiro-OMeTAD, the best hole transport material in perovskite-based solar cells^47^, was employed as the hole extraction/electron blocking layer. Figure 3(b) displays the energy level diagram of the photodetector. The conduction band minimum (CBM, −3.9 V) of TiO_2_ matches the lowest unoccupied molecular orbital (LUMO) level of the peroviskite well, thus, facilitating electron transport, whereas its deep valence band maximum (VBM, −7.2 eV) can block holes effectively. On the contrary, the highest occupied molecular orbital (HOMO) energy level (−5.5 eV) of the perovskite is very close to that of Spiro-OMeTAD (−5.2 eV), which is favorable for hole extraction. Meanwhile, the Spiro-OMeTAD film can block electrons efficiently due to its shallow LUMO energy level (−2.4 eV). Figure 3(c) shows the cross-sectional SEM micrograph of the photodetector. The thickness values of the TiO_2_, α-FAPbI_3_ and Spiro-OMeTAD layers are estimated to be about 45 nm, 400 nm, and 250 nm, respectively. Particularly, the perovskite is thick enough to absorb light efficiently. It is obvious that the α-FAPbI_3_ film is compact and void free. It consists of large-size crystallites that can stretch across the whole photoactive layer, which will lead to efficient carrier transport.

A dark current is the main origin of noise^48^ and is inevitable in a photodetector under biases which limits the ability of detecting weak light signals. Besides, the ratio of the current under light illumination to that in the dark at a bias voltage, namely the on/off current ratio, is an important figure of merit for a photodetector. We evaluated the current density-voltage (*J*-V) characteristics of the FAPbI_3_ photodetector both in the dark and under different intensities of white light illumination, which are exhibited in Fig. 3(d). The bias voltage varied from 1.2 V (forward) to −1.2 V (reverse) that were relatively low for photodetectors, and the white light intensities were selected to be 1 mW/cm^2^, 10 mW/cm^2^, and 100 mW/cm^2^, respectively. In the dark, the current density increases rapidly under forward biases since there is no hole injection barrier from the MoO_3_/Ag anode and a small electron injection barrier of 0.8 eV from the ITO cathode, as shown in Fig. 3b. Under reverse biases, due to the shallow LUMO level of Spiro-OMeTAD and the deep VBM of TiO_2_ forming two Schottky barriers at the electrodes, it is difficult for electrons to be injected from the anode and holes to be injected from the cathode, resulting a very slow rise in the current density. Obviously, the photodetector shows a typical diode-rectifying behavior. Generally speaking, photodiode-type photodetectors operate under reverse bias conditions. The dark current densities of the device at reverse bias voltages of −0.2 V, −0.5 V, and −1.0 V are summarized in Table 1. The dark currents are found to be below the order of magnitude value of 10^−8^ A, and the smallest one is 8.6 × 10^−9^ A at −0.2 V. These results reveal that the dark currents in our device are suppressed dramatically by using TiO_2_ at the cathode and Spiro-OMeTAD at the anode to prevent carrier injections. Under illumination, the photocurrent density increased remarkably with the intensity of white light at the same reverse bias voltage, indicating a strong capability of converting a light signal into an electrical one of the device. Another apparent result is that the photocurrent density remains almost unchanged as the reverse bias voltage increases under the same light irradiance. This is reasonable considering the fact that the photo-generated current is extremely larger than the dark current so that the slight increase of the dark current with the reverse bias voltage can neglected. The photocurrent densities at −0.2 V, −0.5 V, and −1.0 V under white light illumination of 100 mW/cm^2^ are almost the same (16.3–16.5 mA/cm^2^), also shown in Table 1. Under illumination, the perovskite layer can absorb photons with energies larger than the bandgap of α-FAPbI_3_ and generate electron-hole pairs that subsequently dissociate at theTiO_2_/FAPbI_3_ and FAPbI_3_/Spiro-OMeTAD interfaces. Due to the perfect energy level alignment, the separated holes and electrons can effectively transport through the Spiro-OMeTAD and TiO_2_ layers, and then collected by the anode and the cathode, respectively, generating a huge photocurrent in the device^49^. Since the device is reversely biased, the applied electric field is beneficial to enhancing the dissociation and transport processes and reducing the recombination probability of carriers^37^. Consequently, on/off current ratios as high as 10^4^ (see Table 1) have been achieved in the photodetector at different voltages, comparable with the one reported for the CH_3_NH_3_PbI_3_ photodetectors^39^.

**Table 1:** Figures of merit parameters for the photodetectors under different biases.

Voltage (V)	*J*_dark_ (mA/cm2)	*J*_photo_ (mA/cm^2^)	On/off Ratio	*R*(A/W)	D* (Jones)
−0.2	1.9 × 10^−4^	16.3	8.6 × 10^4^	0.33	1.4 × 10^12^
−0.5	2.2 × 10^−4^	16.4	7.5 × 10^4^	0.65	2.4 × 10^12^
−1.0	3.7 × 10^−4^	16.5	4.5 × 10^4^	0.95	2.8 × 10^12^

*J*_photo_ and on/off current ratio were measured under 1 sun AM 1.5 G broadband illumination. *R* and D* were evaluated under 650 nm illumination (5.9 μW/cm^2^).

**Figure 4:**
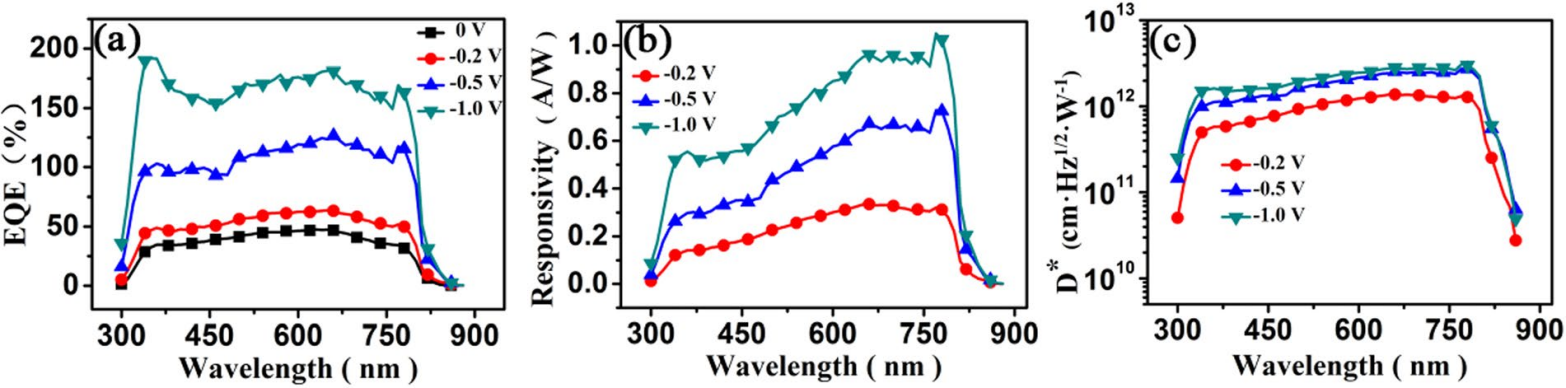
(**a**) External quantum efficiency, (**b**) photoresponsivity, and (**c**) specific detectivity of the photodetector at different reversed bias voltages.

External quantum efficiency (EQE), which relies on the photoactive material and device configuration, is used to assess the photoelectric conversion capability of a phtodetector^49,50^. EQE is defined as the number of photo-generated carriers that an incident photon at a given wavelength can produces per second, and can be calculated by the expression:

2 $$EQE=\frac{{J}_{ph}h\upsilon }{{P}_{in}e}=\frac{({J}_{light}-{J}_{dark})h\upsilon }{{P}_{in}e}$$

where *J*_*ph*_ is the photocurrent density, *P*_*in*_ is the incident light intensity, *e* is the absolute value of electron charge, *J*_*dark*_ is the dark current density, and *J*_*light*_ is the current density under light illumination. We analyzed the EQE value as a function of the wavelength of the incident light of the photodetector under different reverse biases, which are depicted in Fig. 4(a). The light intensity spectrum for the EQE test is shown in Figure S3. The device presents nearly flat EQE spectra spanning the n-UV to NIR regions regardless of whether it is biased reversely, manifesting its similar photoelectric conversion capability for light at a wavelength ranging from 330 to 800 nm. Although weaker light absorption of the photoactive α*-*FAPbI_3_ layer is observed at longer wavelengths, this result can be interpreted by the increased number of incident photons originating from lower photon energies at longer wavelengths. The EQE values of the device enhances gradually as the bias voltage changes from 0 V to −1.0 V and exceed 100% at a voltage greater than −0.5 V, demonstrating that carriers are also injected by the electrodes under an applied bias besides the photo-generated ones^39,40^. The maximum EQE value of about 182% has been achieved at −1.0 V under 650 nm light illumination. Apparently, the operating mechanism of our photodetector is related to the device structure, and the TiO_2_ nanocrystal layer is believed to play a critical role. In addition to the fact that the applied electric field promotes the dissociation of photo-generated electron-hole pairs and the transport of the photo-generated carriers and reduces the carrier recombination^49^, the following two factors should be taken into account for the explanations of the operating mechanism of the device. Under illumination, on the one hand, some photo-generated electrons are trapped in the TiO_2_ layer, and might form charge accumulation and result in enhanced hole injection^38^. On the other hand, the work function of TiO_2_ nanocrystal film is reported to be reduced as the occupation of the deep trapping sites increases after light excitation. Similarly, the trapping sites in the TiO_2_ nanocrystal layer may be occupied by some photo-generated electrons from the perovskite layer, leading to a decrease in the work function of the TiO_2_ film, which probably also facilitates the injection of holes from the cathode^45,51^.

Photoresponsivity *R* and specific detectivity D* are key performance figure of merits in photodetectors. *R* is the ratio of the photocurrent to the intensity of the incident monochromatic light, which can be estimated by the following equation:

3 $$R=\frac{{J}_{ph}}{{P}_{in}}=\frac{EQE\cdot e}{h\upsilon }$$

D* is the ability of a photodetector to detect weak light signals. When the dark current is dominated by shot noise^48^, the specific detectivity is determined by the formula:

4 $${D}^{\ast }=\frac{R}{\sqrt{2e{J}_{dark}}}$$

We derived the photoresponsivity spectra of the photodetector at the bias voltages of −0.2 V, −0.5 V, and −1.0 V based on the EQE spectra since they are closely correlated, which are plotted in Fig. 4b. The device exhibits a broad spectral response from the n-UV (330 nm) to the NIR regions (800 nm) attributed to the broadband absorption of the α-FAPbI_3_ film, wider than that of the photodetectors based on MAPbI_3_ (350–750 nm)^49^. The photoresponsivity at each bias increases with the increasing of the wavelength of incident light, and reaches a maximum at 650 nm and remains unchanged up to 800 nm. In addition to showing a positive relationship with EQE, photoresponsivity is inversely proportional to the incident photon energy, thus, the photodetector is more sensitive in detecting longer-wavelength light signals. It is also evident that the photoresponsivity increases as the reverse bias goes up at the same wavelength, showing a similar trend to the EQE value. At −1.0 V, the photoresponsivity can achieve 0.95 A/W at 650 nm light illumination. The values of the specific detectivity were evaluated according to Equation (4). Figure 4(c) displays the spectra of the specific detectivity at bias voltages of −0.2 V, −0.5 V, and −1.0 V, respectively. There is no doubt that the detectivity possesses exactly the same spectral response range. At the same bias the change of the detectivity with the incident wavelength resembles the one of the photoresponsivity, while at the same wavelength the change of the detectivity with the reverse bias is influenced by the dark current of the device as well. The values of the specific detectivity are above 10^12^ Jones (cm Hz^1/2^ W^−1^) from 330 to 800 nm at the reverse bias of −0.5 V and a peak value of 3.1 × 10^12^ Jones is obtained at 770 nm at −1.0 V. The high detection capability of our device can be mainly ascribed to the suppressed dark current coming from the device structure. The photoresponsivity and specific detectivity values of the device at different biases are summarized in Table 1 along with the dark and photocurrent densities and the on/off current ratios.

**Figure 5:**
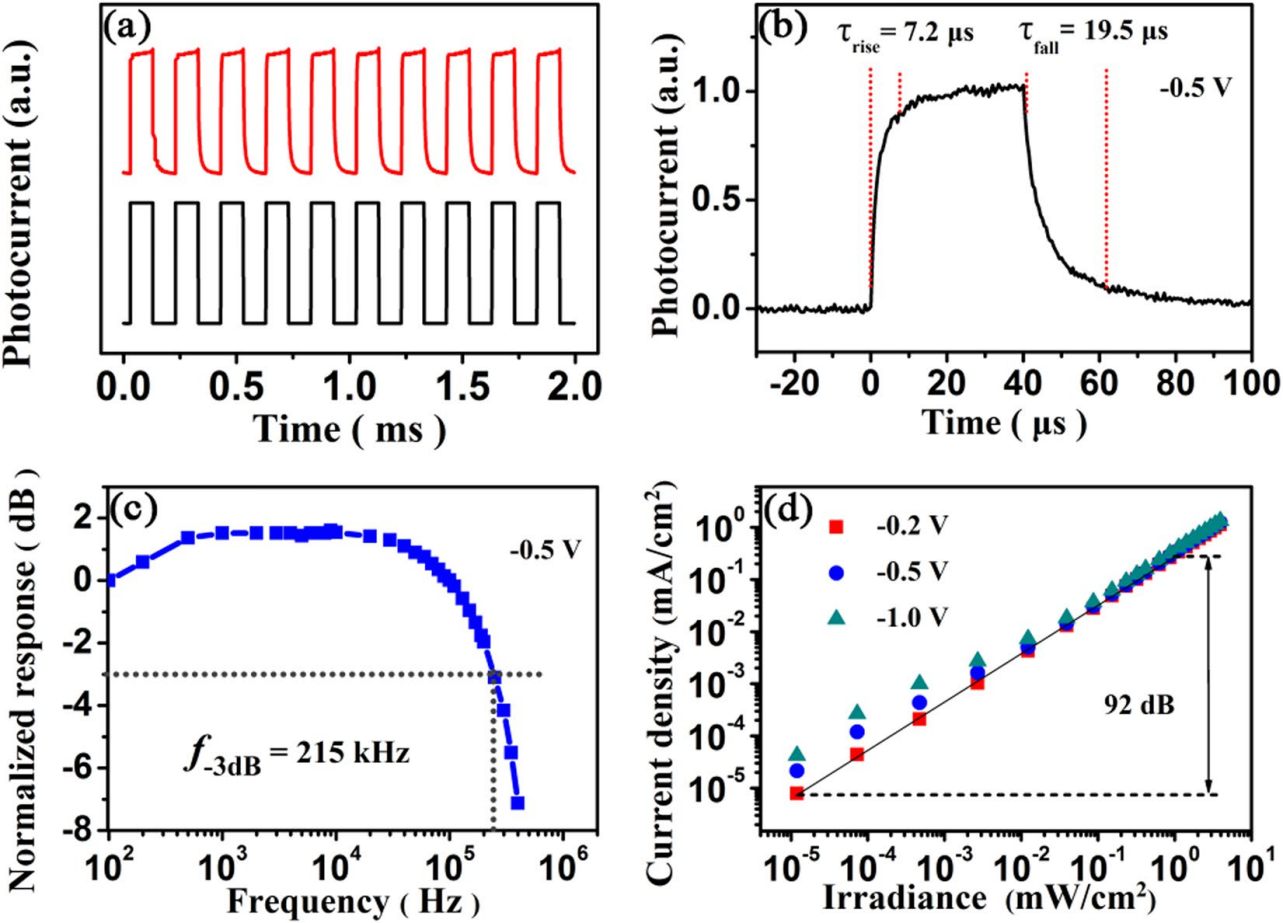
(**a**) Transient photocurrent at a frequency of 5000 Hz, (**b**) single normalized cycle of the photocurrent at a frequency of 500 Hz, and (**c**) frequency response of the photodetector at−0.5 V under modulated green-LED (530 nm) illumination. (**d**) Photocurrent density as a function of light intensity of the photodetector under green-LED (530 nm) illumination at different reversed bias voltages.

Other parameters, such as the temporal response and photosensitivity linearity, are also important in evaluating photodetectors. The temporal response of a photodetector is characterized by response rise and fall times or a response bandwidth. The transient photocurrent of the device at the bias of −0.5 V was measured under a 530 nm pulse light from a light-emitting diode (LED) with a duration of 0.1 ms at a repeating frequency of 5000 Hz generated by a function generator, as illustrated in Fig. 5(a). With the excitation light on and off, the transient photocurrent curve shows fast rise/fall processes and good repeatability during the 10 cycles of measurements. To evaluate the rise and fall times accurately, which are defined as the times taken for a photodetector to reach 90% and drop to 10% of steady-state values respectively^50^, the temporal response of the photocurrent under the pulse light with a shorter duration (40 μs) at 500 Hz was investigated, as plotted in Fig. 5(b). The device shows a rise time of 7.2 μs and a fall time of 19.5 μs, which are close to those (20/17 μs) of the MAPbI_3_ thin film photodetectors^37^. Both the rise time and fall time are limited by charge trapping and detrapping processes since charge generation and carrier drifting processes should be much faster. The fall time of the device is longer than the rise one because of the influence of the charge detrapping as explained in the literature^40^. The −3 dB bandwidth is the frequency of light signal at which the photocurrent is 70.7% of that under steady light^52^. To obtain the bandwidth of the photodetector, the small signal frequency response at −0.5 V under modulated 530 nm illumination was investigated, and the photocurrents have been normalized to the value measured at 100 Hz, as illustrated in Fig. 5(c). The bandwidth of the device is estimated to be 215 kHz.

Lastly, to express the photosensitivity linearity of the device^49^, a range within which the photocurrent shows a linear response to the incident light intensity, the photocurrent density versus the incident light intensity curves were measured under green-LED (530 nm) illumination with the light intensity varying from 12 nW/cm^2^ to 3.9 mW/cm^2^ at different reverse biases. Figure 5(d) shows linear responses of the photodetector at −0.2 V, −0.5 V, and −1.0 V. It can be seen that the photocurrent density increases linearly with increasing the light intensity, and the photodetector has better linearity at −0.2 V. The linear dynamic range at −0.2 V is estimated to be 92 dB. In addition, the slight deviations from linearity are also observed in the lower light intensity region from nW/cm^2^ to μW/cm^2^ as the bias voltage increases. This is understandable because under weak light illumination the injected charges from the electrodes make greater contributions to the photocurrent density compared with the photo-generated charges, while they can be neglected under strong light.

## Conclusions

In summary, we have used a one-step solution processing method to prepare FAPbI_3_ thin films, and obtained pure polycrystalline α-FAPbI_3_ films by annealing at a temperature of 150 °C. We have demonstrated high-overall-performance photodetectors based on the α-FAPbI_3_ films. The photodetectors, exhibiting a broadband response from the n-UV to the NIR (330–800 nm), can operate at a bias voltage as low as −0.2 V with a low dark current density of 8.6 × 10^−9^ A and a high on/off current ratio of 8.6 × 10^4^. The EQE of the devices becomes bigger than 100% at −0.5 V and reaches 182% at −1.0 V under 650 nm light illumination. The devices yield a photoresponsivity of 0. 95 AW^−1^ and a high specific detectivity of 2.8 × 10^12^ Jones at −1.0 V under 650 nm illumination with fast response times of 7.2/19.5 μs. Our results have proved that polycrystalline α-FAPbI_3_ is an excellent photoactive material for photoconductors, and the photodiode-type photodetectors based on the α-FAPbI_3_ thin films with superior performance show great promise for future optoelectronic device applications.

## Methods

### Material preparation

Lead iodide (PbI_2_, 99%) and formamidinium iodide (FAI, 99%) were purchased from Alfa Aesar and Dyesol, respectively. 2,2′,7,7′-Tetrakis[N,N-di(4-Methoxyphenyl)aMino]-9,9′-spirobifluorene (Spiro-OMeTAD, 99%) was obtained from Nichem, and lithium salt (Li-TFSI, 99%) and 4-tert-butylpyridine (TBP, 96%) were supplied by Sigma-Aldrich. TiCl_3_ (15.0–20.0% basis in 30% HCl) and ethyl alcohol (98%) were purchased from Aladdin. Dimethyl formamide (DMF, 99.8%) and chlorobenzene (CB, 99.5%) were supplied by Alfa Aesar. All materials were used as received without further purification. 1 M precursor solutions of FAPbI_3_ were prepared by stoichiometric amounts of PbI_2_ and FAI dissolved in DMF, and then stirred at 70 °C for 12 hours. A solution of Spiro-OMeTAD was prepared by adding 68 mg of Spiro-OMeTAD, 17.5 μl of lithium salt stocking solution (500 mg Li-TFSI in 1 ml acetonitrile) and 28 μl of TBP in 950 μl. The ligand-free anatase TiO_2_ nanocrystals were synthesized from an aqueous solution of TiCl_3_ (20%, 1 mL) and HCl (6 M, 1.0 mL) mixed with ethyl alcohol (60 mL) at low temperatures through a hydrolytic sol–gel reaction, as reported in our previous work^44,46^, and dispersed into deionized water (20 wt%). Films of FAPbI_3_ were deposited onto precleaned glass substrates from the precursor solutions of 50 μl at 3000 rpm for 15 sec with a very small amount of toluene as an anti-solvent during deposition, then dried at 5000 rpm for 30 sec, and last annealed in a nitrogen-filled glovebox at temperatures from 70 °C to 150 °C for 20 min.

### Device fabrication

The ITO-coated glass substrates with a sheet resistance of 15 Ω/square were firstly cleaned with detergent, and ultrasonicated in deionized water, acetone, and isopropyl alcohol for 30 min each, and then subsequently dried by a nitrogen flow. The solution of TiO_2_ nanocrystals was spin coated onto the cleaned ITO substrate at 2000 rpm for 50 s in ambient conditions, and then annealed in air at 100 °C for 10 min and in nitrogen atmosphere at 130 °C for 10 min, respectively, forming an electron extraction layer. The substrate coated with the TiO_2_ layer was then transferred into a nitrogen-filled glove box. A film of FAPbI_3_ was deposited onto the TiO_2_ layer from the precursor solution of 50 μl at 3000 rpm for 15 sec with a very small amount of toluene as an anti-solvent during deposition, and then was dried at 5000 rpm for 30 sec. The substrate with the films of FAPbI_3_ and TiO_2_ was subsequently annealed in the glove box at 150 °C for 20 min. In the next step, a hole extraction layer of Spiro-OMeTAD was spin coated on the perovskite film at 5000 rpm for 50 s, followed by drying at ambient temperature. Finally, 9-nm-thick film of molybdenum trioxide (MoO_3_) and 100-nm-thick silver (Ag) electrode was thermally deposited onto the hole extraction film through a shadow mask in a vacuum chamber with a pressure of 10^−7^ mbar. The effective illumination area of the photodetector is 4.5 mm^2^.

### Material and device characterizations

The XRD patterns were detected with a Bruker D8 Advance X-ray diffractometer. The morphologies of the TiO_2_ film and the FAPbI_3_ films were characterized using a Shimadzu SPM-9700 atomic force microscope (AFM) and a Hitachi S-4800scanning electron microscope (SEM), respectively. The absorption spectra of the photoactive perovskite layers were measured by an ultraviolet-visible (UV-vis) absorption spectrophotometer (UV-3101PC, Shimadzu). The photoluminescence (PL) spectra of the FAPbI_3_ films were recorded by a Fluorolog (Horiba) spectrofluorometer. An AM 1.5 solar simulator (ABET Technologies) at 100 mW/cm^2^ intensity was used to provide white light illumination. The Current density versus voltage (*J*-V) characteristics of the photodetector in the dark and under illumination were investigated with a Keithley 6430 source-power unit. The external quantum efficiencies (EQE) of the device were recorded in a solar cell QE/IPCE measurement system (Zolix Solar Cell Scan 100) with a Keithley 2450 source meter providing a suitable bias voltage. The transient photocurrents were measured by the photodetector connected with a resistor of 50 Ω in series and the voltage applied to the resistor collected by an oscilloscope (Tektronix, MSO-5104B) under a square-pulsed optical excitation that was generated from a 530-nm light emitting diode (LED) driven by a function generator. The response bandwidth of the photodetector was calculated with fast Fourier transform (FFT) algorithm. All measurements were carried out at room temperature under ambient conditions.

## Electronic supplementary material

Supplementary Information
