# Supplementary material for: High-Performance Photodiode-Type Photodetectors Based on Polycrystalline Formamidinium Lead Iodide Perovskite Thin Films
Source: Sci Rep. 2018 Jul 24;8:11157. doi: 10.1038/s41598-018-29147-6 (PMC6057967; doi:10.1038/s41598-018-29147-6)
Supplement: Supplementary file 1 — Supplementary Information [file 41598_2018_29147_MOESM1_ESM.pdf]

# Supplementary Information

# High-Performance Photodiode-Type Photodetectors Based on Polycrystalline Formamidinium Lead Iodide Perovskite Thin Films

*Meng Zhang, Fan Zhang, Yue Wang, Lijie Zhu, Yufeng Hu*, Zhidong Lou*, Yanbing Hou, and Feng Teng*

Key Laboratory of Luminescence and Optical Information, Ministry of Education, Institute of Optoelectronic Technology, Beijing Jiaotong University, Beijing, 100044.

*Corresponding author: E-mail: yfhu@bjtu.edu.cn and zhdlou@bjtu.edu.cn

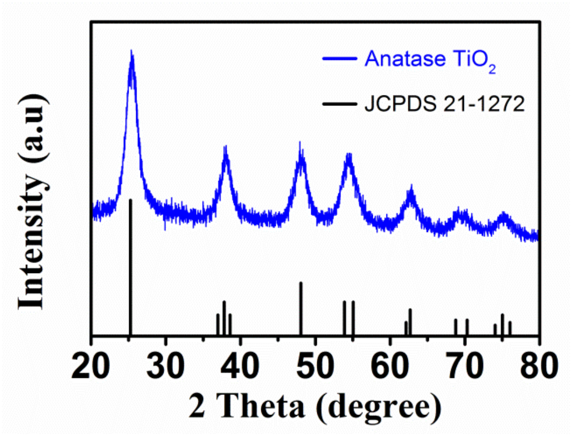


Figure S1. XRD patterns of the $TiO_2$ nanocrystals.

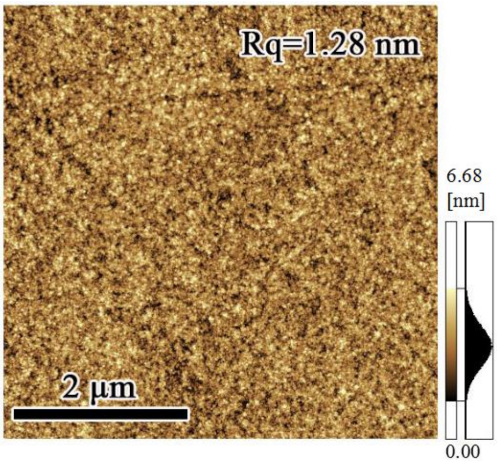


Figure S2. AFM image of the $TiO_2$ nanocrystal film.

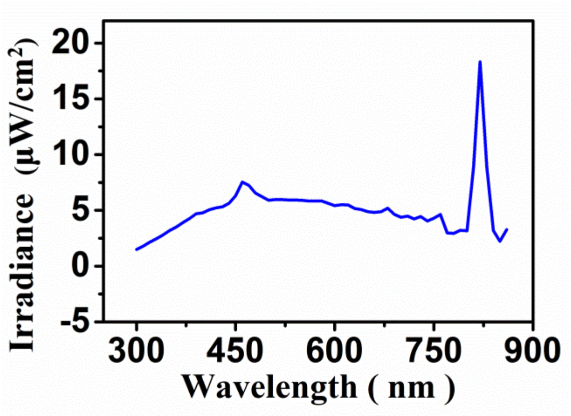


Figure S3. Light intensity spectrum for the EQE test of the perovskite photodetector.
